# Inhibition of microRNA-139-5p by glucagon-like peptide-1 ameliorates oxidative stress-induced vascular endothelial cell damage via targeting SOD1/GCLc

**DOI:** 10.1530/EC-25-0022

**Published:** 2025-04-29

**Authors:** Jiaqi Zhang, Jiake Mo, Ying Liu, Xubiao Meng, Weian Tang, Lanfang Fu, Jing Xiong, Zhaohui Mo

**Affiliations:** ^1^ Department of Endocrinology, The Third Xiangya Hospital of Central South University, Changsha, Hunan Province, China; ^2^ Diabetic Foot Research Center of Central South University, Changsha, Hunan Province, China; ^3^ Department of Endocrinology, Haikou People’s Hospital & Haikou Affiliated Hospital of Central South University Xiangya School of Medicine, Haikou, Hainan Province, China

**Keywords:** glucagon-like peptide-1, vascular endothelial cells, microRNA-139-5p, oxidative stress, superoxide dismutase-1, glutamate-cysteine ligase

## Abstract

**Graphical abstract:**

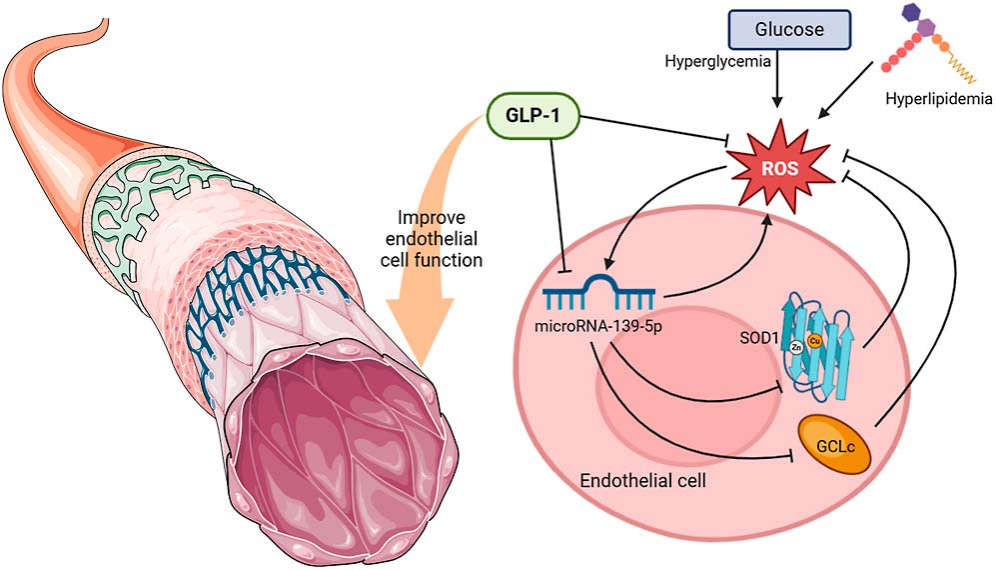

**Abstract:**

Oxidative stress is a key driving factor for the progression of vascular disease in diabetes, and is closely related to endothelial dysfunction. The exact mechanism by which glucagon-like peptide-1 (GLP-1) directly protects vascular endothelium by reducing oxidative stress is not yet fully understood. In this study, we investigated the protective effect of GLP-1 on endothelial cells exposed to palmitic acid (PA)/high glucose-induced oxidative stress and further explored the potential mechanisms involved in microRNA-139-5p (miR-139-5p) regulation. We found that miR-139-5p expression was exhibited significantly elevated in HUVECs that were exposed to PA/high glucose or H_2_O_2_, which were reversed by glutathione. Interestingly, this expression was significantly attenuated after GLP-1 pretreatment, with reduced reactive oxygen species (ROS), increased GSH/GSSG ratio and amelioration of cell dysfunction. Overexpression of miR-139-5p resulted in increased ROS and apoptosis, decreased GSH/GSSG ratio, damaged migration and proliferation of HUVECs, while inhibition of miR-139-5p significantly restored PA-induced HUVECs impairments. Further investigation revealed that miR-139-5p directly targets superoxide dismutase 1 (SOD1)/glutamate–cysteine ligase catalytic (GCLc) subunit. The upregulation of miR-139-5p abrogated the protective effects of GLP-1 on cells exposed to PA, and GLP-1-induced downregulation of miR-139-5p was counteracted by the GLP-1 receptor antagonist exendin(9–39). These findings demonstrated that GLP-1 ameliorates oxidative stress-induced endothelial dysfunction, at least in part, by suppressing miR-139-5p, which targets SOD1 and GCLc. This provides further evidence for the vascular protective effects of GLP-1 intervention in diabetes.

## Introduction

Diabetes mellitus has become one of the most prevalent diseases causing high disease burden worldwide. The number of patients with diabetes globally will reach an estimated 643 million by 2030, and nearly 6.7 million people died as a result of diabetes and its complications in 2021 (1). Diabetic vascular complications, both macroangiopathy and microangiopathy, are the primary causes of death and disability in individuals with diabetes (2). Type 2 diabetes mellitus (T2DM) accounts for over 90% of all diabetes cases. Intensive glucose lowering has proven to be effective in reducing microvascular disease in patients with T2DM. However, its impact on cardiovascular events and mortality is still a subject of diabetes. As a result, there is an increasing need for new antidiabetic drugs that not only reduce blood glucose levels but also offer cardiovascular protection.

Glucagon-like peptide-1 (GLP-1), an incretin hormone released by intestinal L cells, boosts cellular glucose uptake by promoting insulin secretion and enhancing insulin sensitivity in target tissues (3). Initially developed as an antidiabetic drug, GLP-1 has garnered significant attention in recent years due to its demonstrated cardiovascular protective effects (4). Clinical trials and animal experiments have demonstrated that both native GLP-1 and pharmacological GLP-1 receptor agonists (GLP-1 RAs) decrease the risk of cardiovascular events in individuals with T2DM (5, 6) through mechanisms such as reducing blood pressure, improving endothelial function and decreasing inflammation (7). While the underlying mechanisms remain incompletely understood, alleviating oxidative stress is believed to play an important role in the cardiovascular protection afforded by GLP-1 analogs (8).

Oxidative stress is a key driver in diabetic vascular disease progression, closely linked to endothelial dysfunction (9). Under normal conditions, the body maintains redox homeostasis by regulating the balance between oxidative and antioxidant systems. However, in diabetic vascular endothelial cells (ECs), there is often an abnormal increase in nicotinamide adenine dinucleotide phosphate oxidase (Nox) activity (10), xanthine oxidase (11) and protein kinase C (PKC) activation (12), leading to elevated levels of reactive oxygen species (ROS). The Kelch-like ECH-associated protein 1 (Keap1)-nuclear factor erythroid 2-related factor 2 (Nrf2) signaling pathway, a key defense system, and its target genes, including superoxide dismutase 1 (SOD1), heme oxygenase-1 (HO-1) and glutamate–cysteine ligase catalytic subunit (GCLc), are impaired in ECs exposed to high glucose (HG) levels (13) and in the retinas of donors with diabetic retinopathy (14). However, the exact mechanism underlying dysfunction of these defense systems in ECs of patients with diabetes remain unclear. GLP-1RAs may directly protect vascular endothelium through reducing oxidative stress (15). One study showed that GLP-1 treatment can protect ECs from autophagy induced by oxidative stress (16). Nevertheless, the exact mechanisms by which GLP-1 improves oxidative stress-induced endothelial dysfunction are not fully understood.

MicroRNAs, a group of small endogenous RNAs, play a crucial role in regulating gene expression post-transcriptionally (17). In recent years, it has been observed that dysregulated microRNAs can directly or indirectly affect antioxidant signaling pathways (18), such as Keap1/Nrf2 (19), sirtuin 1 and forkhead box O, and effector enzymes such as SOD1/2 and HO-1. MicroRNA-139-5p (miR-139-5p) has drawn particular interest due to its involvement in regulating genes related to ROS defense pathway in tumor cells (20) and its role in the pathogenesis of diabetes, specifically in endothelial progenitor cells (EPCs) (21). Our previous study found an association between upregulated miR-139-5p and vascular endothelium dysfunction in diabetes (22). Another study (23) discovered that the reduction in miR-139-5p expression is linked to the anti-apoptotic effects of liraglutide on pancreatic and INS-1 cells in diabetic rats by targeting insulin receptor substrate-1 (IRS1). Nevertheless, the involvement of miR-139-5p in oxidative stress-induced EC damage and its contribution to the endothelial protective effect of GLP-1 remain to be elucidated.

In this study, we investigated the protective effect of GLP-1 on ECs exposed to palmitic acid (PA)/HG-induced oxidative stress, and delved deeper into the underlying mechanisms involving the regulation of miR-139-5p. By elucidating the role of miR-139-5p in oxidative stress-induced EC damage and its association with the protective effect of GLP-1, we hope to provide insights into potential therapeutic strategies for preventing endothelial dysfunction in diseases such as diabetes.

## Methods

### Cell culture and treatment

Human umbilical vein endothelial cells (HUVECs) were purchased from the Shanghai Cell Bank of the Chinese Academy of Sciences (CAS, China) and cultured in Roswell Park Memorial Institute (RPMI) 1640 medium (Procell, China) containing 10% fetal bovine serum (FBS) (Procell, China) at 37°C in a humidified atmosphere with 5% CO_2_. For the construction of *in vitro* oxidative stress model, HUVECs were exposed to PA (Solarbio, China) at a final concentration of 100 μM or HG (33 mM) within RPMI 1640 and 10% FBS, or induced by hydrogen peroxide (H_2_O_2_) for a final concentration of 200 μM. Reduced glutathione powder (GSH) (MeilunBio, China) was dissolved in sterile aqueous solution and preincubated at a final concentration of 5 mM for 1 h before H_2_O_2_ exposure.

### GLP-1(7–37) acetate treatment, exendin-4, exendin(9–39) and experimental settings

GLP-1(7–37) acetate, Cat. No: HY-P0055A. and exendin(9–39), Cat. No: HY-P0264. were purchased from MCE Biologicals (MedChemExpress, USA). GLP-1 powder was dissolved in a sterile aqueous solution containing acetic acid and filtered through a sterile filter (0.22 μm) (Millipore, USA) to create a 0.1 mg/mL GLP-1 solution. The exendin(9–39) solution (2 mM) was prepared by dissolving the powder in sterile water and filtering through a 0.22 μm membrane. Exendin-4 was purchased from APE (APExBIO Technology, USA), which was formulated according to the same method as for GLP-1. To investigate the protective effects and mechanisms of GLP-1 in HUVECs exposed to PA, cells were seeded at 5,000 cells/cm^2^ on 6-well plates. Oxidative stress was induced by exposing HUVECs to 100 μM PA or 33 mM HG, and GLP-1 was administered 2 h before PA exposure by incubating in RPMI 1640 containing 0.5% FBS. In accordance with the pre-experiments, the final concentrations of GLP-1 (50 nM), exendin-4 (20 nM) and exendin(9–39) (50 nM) were selected for application in the subsequent experiments, respectively.

### MicroRNA transfection

MicroRNA transfection was conducted by Lipofectamine™ 3,000 (Invitrogen, USA), following the manufacturer’s guidelines. The miR-139-5p mimic, miR-139-5p inhibitor, negative control of miRNA mimic/inhibitor (mimic-NC/inhibitor-NC) were chemically synthesized by RiboBio (China). HUVECs were seeded into 6-well plates at a density of 1 × 10^5^ cells per well and cultured for 24 h before transfection. After 48 h post-transfection, the HUVECs were subjected to further studies.

### Cell migration capacity assay

HUVECs were grown on 6-well plates until confluent. The monolayer was then scraped with a 10 μL pipette tip. After gentle washing with phosphate buffer saline (PBS), the cells were switched to low serum medium. The scratches at 0 and 24 h were recorded with a normal optical microscope (100×) (Leica MC170HD, Germany). Cell migration distance was quantified using the ImageJ 1.53a (NIH, USA).

### Cell counting kit 8 (CCK8) assay

HUVECs were inoculated on 6-well culture plates and treated accordingly. Different groups of HUVECs were added to 96-well plates with a density of 3,000 cells/well and cultured for 24 h. After the initial incubation, the medium was aspirated and replaced with 90 μL serum-free medium in each well, added 10 μL CCK8 reagent (Dojindo, Japan). Control wells containing only medium and CCK8 reagent without cells were included to account for background absorbance. After an additional 2 h incubation, the absorbance was measured at 450 nm.

### Apoptosis detection by flow cytometry

HUVECs were harvested in accordance with the manufacturer’s directions and rinsed twice with ice-cold PBS. Subsequently, the cells were resuspended in 500 μL binding buffer (Next Sage Biotechnology, China) containing Annexin V-Alexa Fluor 647 (5 μL) and PI (10 μL) and incubated for 15 min at 37°C away from light. The samples were acquired and analyzed using the BD FACSVerse™ (BD Biosciences, USA). Annexin V-Alexa Fluor 647-positive but PI-negative cells were identified as early apoptotic, while double-stained cells were classified as late apoptotic or necrotic. The total apoptosis rate was quantified by summing the percentages of early apoptotic and late apoptotic cells.

### Cellular ROS detection

Cellular ROS levels were measured using a ROS assay kit (Beyotime, China). HUVECs were seeded into 6-well culture plates and cultivated in serum-free medium containing 2′,7′-dichlorodihydrofluorescein diacetate (DCFH-DA) (10 μM) (15 min, 37°C in the dark). Then, cells were gently washed with PBS and observed the fluorescent product 2′,7′-dichlorofluorescein (DCF) using a fluorescent microscope (Leica MC170HD, Germany). The quantification of ROS was performed by measuring fluorescence intensity in three random fields per group using the ImageJ software (NIH, USA).

### Measurement of GSH and GSSG

GSH and GSSG levels in HUVECs were determined using commercial kits (BOXBIO, China), according to the manufacturer’s protocols. GSH reacts with 5,5′-dithiobis-(2-nitrobenzoic acid) (DTNB) to generate 2-nitro-5-thiobenzoic acid (TNB), a yellow chromophore with a characteristic absorbance peak at 412 nm, allowing for the quantification of GSH based on absorbance changes. To selectively measure GSSG levels, endogenous GSH was first masked using a GSH scavenger, followed by the reduction of GSSG to GSH via glutathione reductase. The regenerated GSH then reacted with DTNB, producing TNB, which was subsequently quantified at 412 nm to determine GSSG concentration. The GSH/GSSG ratio was calculated accordingly to reflect cellular redox balance.

### Dual luciferase reporter assays

The synthetic SOD1 wild-type (WT) and mutant (MUT) or GCLc WT and MUT in pmir-GLO luciferase plasmid were co-transfected respectively with miR-139-5p mimics or NC mimic into HUVECs. After 48 h, the cell lysates were harvested, dual luciferase reporter assays were performed with a Double-Luciferase Reporter Assay Kit (Transgen Biotech, China). Firefly/Renilla luciferase activity were measured using the BioTek Synergy H1 (Agilent, USA) and BioTek GEN5 software (Agilent, USA).

### Angiogenesis (tube formation) assay *in vitro*

The angiogenic capability of HUVECs was determined by matrigel tube formation assay. Cells were pretreated with/without exendin(9–39) for 30 min before GLP-1 intervention for 2 h in palmitate/normal condition, respectively, and then seeded on matrigel matrix (Corning, USA) to form tubes. Tubes were allowed to form for 6 h at 37°C and 5% CO_2_ in a humidified chamber. Images were captured by a Leica MC170HD (Germany) and analyzed using the ImageJ (NIH, USA).

### RNA extraction and quantitative polymerase chain reaction (qPCR)

Total RNA was extracted from HUVECs by TRIzol (Transgen Biotech, China). miRNAs reverse transcription was conducted via miRNA 1st-strand cDNA synthesis kit (Vazyme, China) in conjunction with the Bulge-Loop hsa-miR-139-5p or U6 Primer Set (RiboBio, China). qPCR analyses were performed using SYBR green PCR kit (TOYOBO, Japan) and LightCycler® 480 II PCR system. Relative fold changes of miR-139-5p were normalized to U6 using the 2^−ΔΔCt^ method. Primers for miR-139-5p were 5′ACA​CTC​CAG​CTG​GTC​TAC​AGT​GCA​CGT​GTC3′ (forward); 5′TGG​TGT​CGG​TGG​AGT​CG-3′ (reverse). Primers used for U6 were 5′CTC​GCT​TCG​GCA​GCA​CA-3′ (forward); 5′-AAC​GCT​TCA​CGA​ATT​TGC​GT-3′ (reverse).

### Western blot

Cells were lysed with radioimmunoprecipitation assay buffer containing phenylmethanesulfonyl fluoride (Thermo Fisher Scientific, USA). Protein concentrations in solution were quantified using a bicinchoninic acid protein assay kit (Beyotime, China). Equivalent amounts of proteins (10–20 μg/lane) were loaded onto a 10% sodium dodecyl sulfate polyacrylamide gel and transferred onto a 0.22 μm polyvinylidene fluoride membrane (Millipore, USA). Nonspecific proteins on the membrane were blocked with TBST (0.1% Tween-20 in Tris-buffered saline) containing 5% skimmed milk for 60 min at room temperature. Membranes were incubated overnight at 4°C with the following primary antibodies: anti-B cell lymphoma 2 (Bcl-2) (1:2,000), anti-Bcl-2-associated X protein (Bax) (1:1,000), anti-GCLc (1:2,000), anti-SOD1 (1:2,000), anti-β-tubulin (1:2,000) and anti-β-actin (1:2,000). All primary antibodies were sourced from Proteintech (China). The membranes were then incubated with HRP-conjugated secondary antibodies (Proteintech, China) for 2 h. Immunofluorescent reaction bands were revealed by ECL (Advansta, USA). Statistical comparisons between groups were performed using the Image Lab v.5.1 (Bio-Rad, USA) and Prism 8.1.2, presented as relative ratios.

### Statistical analysis

Statistical analysis of the data was conducted using the GraphPad Prism v8.1.2 (GraphPad, USA). The unpaired Student’s *t*-test was utilized to determine statistical significance between two groups; for multiple comparisons, one-way ANOVA or two-way ANOVA was used and post-hoc analysis was performed with Tukey’s test or Sidak’s test, respectively. Results are presented as the mean ± standard deviation (SD) from a minimum of three independent experiments, and *P* < 0.05 was considered significant.

## Results

### GLP-1 ameliorates oxidative stress damage in HUVECs and downregulates miR-139-5p expression

**Figure 1:**
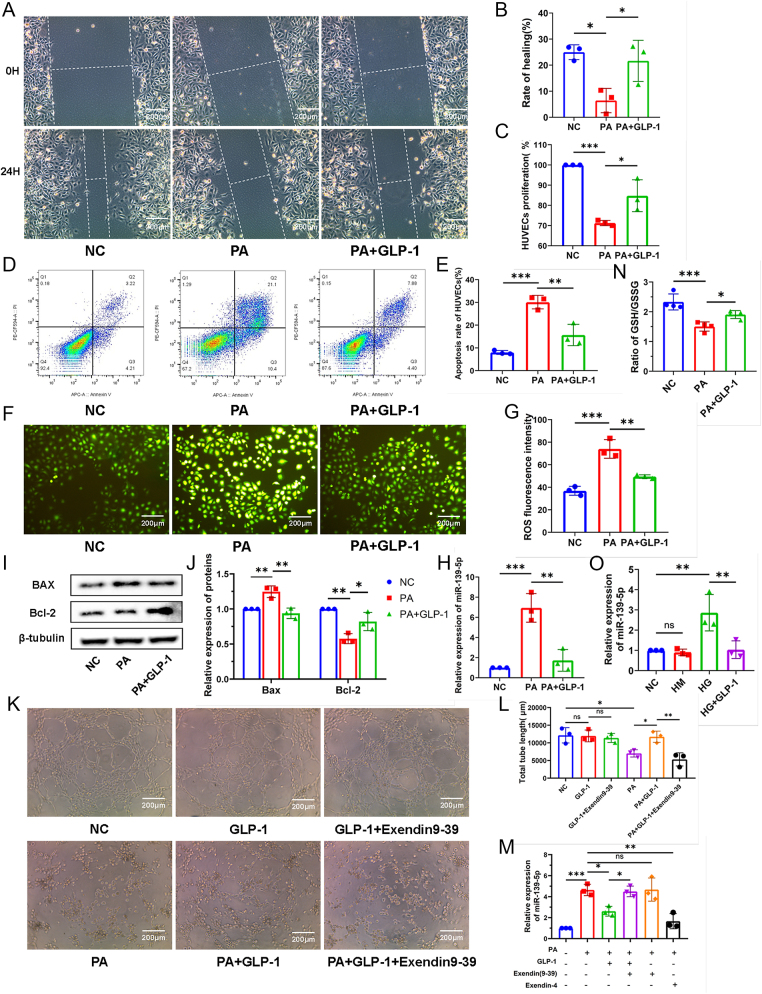
GLP-1 ameliorates oxidative stress-induced damage of HUVECs and downregulates miR-139-5p expression. (A) The effects of GLP-1 on HUVECs migration exposed to PA were evaluated using scratch assay (scale bar: 200 μm). (B) The migration rate was quantified by the ImageJ (*n* = 3). (C) Proliferative effect of GLP-1 on HUVECs exposed to PA was observed by CCK8 kits (*n* = 3). (D) Anti-apoptosis effect of GLP-1 was measured by flow cytometry. (E) Total apoptosis rate was quantified as the sum of early apoptosis and late apoptosis (Q2 + Q3) (*n* = 3). (F) ROS levels in HUVECs exposed to PA with/without GLP-1 were determined by DCFH-DA fluorescence (scale bar: 200 μm). (G) The intensity of ROS fluorescence was measured by the ImageJ (*n* = 3). (H) miR-139-5p expression in HUVECs with PA was determined by qPCR (*n* = 3). (I) Bax and Bcl-2 expression was detected by Western blot, protein levels were normalized using β-actin or β-tubulin and (J) quantified by the Image Lab (*n* = 3). (K) Representative images of tube morphology showing effects of exendin against GLP-1 in normal or PA-treated HUVECs, (scale bar: 200 μm) and (L) quantification of the total tube length measured by the ImageJ (*n* = 3). (M) Expression of miR-139-5p in each group were detected by qPCR, (*n* = 3). (N) Effects of GLP-1 on GSH/GSSG ratio in PA-induced HUVECs (*n* = 4). (O) The expression of miR-139-5p in HG with/without GLP-1 was determined by qPCR (*n* = 3). Data are presented as the mean ± SD. Error bars represent: SDs. *P < 0.05, **P < 0.01, ***P < 0.001, ns *P* > 0.05.

To investigate the protective effect of GLP-1 against oxidative stress-induced endothelial dysfunction, we established oxidative stress-induced damage models using HG or PA treatment. As measured by scratch and CCK8 assays, GLP-1 pretreatment significantly improved the migration and proliferation ability of HUVECs exposed to PA medium (Fig. 1A, B, C). In addition, GLP-1 pretreatment reversed PA-induced increase in cell apoptosis, as measured by flow cytometry (Fig. 1D and E), and significantly ameliorated the alterations in Bcl-2 and Bax levels induced by PA (Fig. 1I and J). The intracellular redox status was assessed using the ratio of GSH to GSSG and ROS levels. Compared with the NC group, HG (Supplementary Fig. 1 (see section on Supplementary materials given at the end of the article)) or PA treatment significantly decreased the GSH/GSSG ratio, whereas GLP-1 pretreatment markedly improved the damaged ratio (Fig. 1N). High ROS levels induced by PA were remarkably reduced when HUVECs were pretreated with GLP-1, indicating that GLP-1 can mitigate oxidative stress (Fig. 1F and G).

Importantly, we found that the expression of miR-139-5p was markedly elevated in HUVECs exposed to PA for 24 h, and this upregulation was also observed under HG conditions. However, GLP-1 pretreatment significantly attenuated the elevated miR-139-5p levels induced by both PA and HG (Fig. 1H and O), while this effect was counteracted by the GLP-1 receptor antagonist exendin(9–39) (Fig. 1M). Meanwhile, the analysis of small tube formation assay showed that exendin(9–39) treatment prevented GLP-1 from improving the tubular formation ability of HUVECs in PA (Fig. 1K and L). In summary, these findings suggest that the protective effect of GLP-1 may involve regulation of miR-139-5p expression.

### miR-139-5p is associated with oxidative stress-induced damage to HUVECs

**Figure 2:**
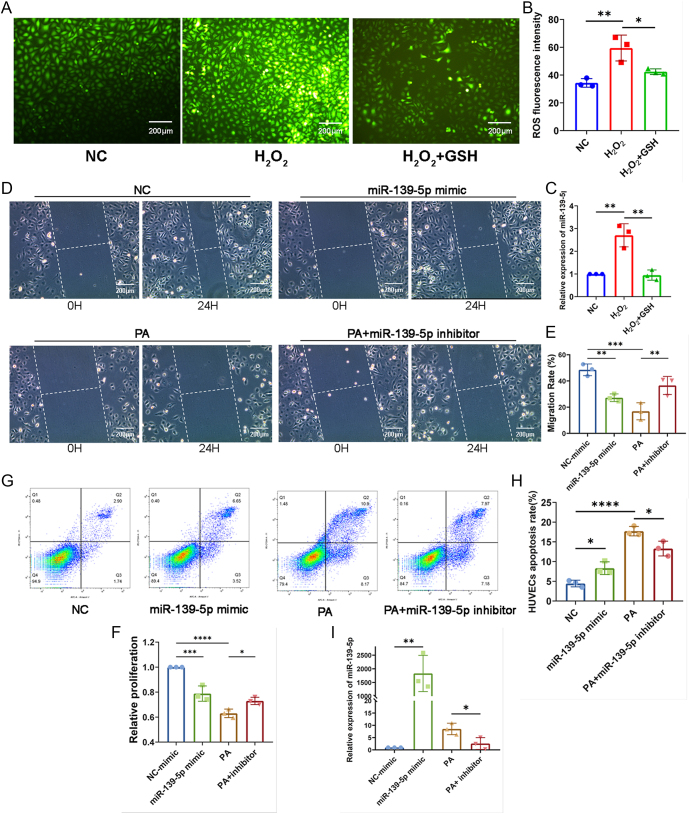
miR-139-5p associated with oxidative stress-induced damage to HUVECs. (A) ROS levels in HUVECs exposed to H_2_O_2_ and treated with GSH were measured using the DCFH-DA fluorescence assay (scale bar: 200 μm). (B) The intensity of ROS fluorescence was quantified by the ImageJ (*n* = 3). (C) miR-139-5p expression in HUVECs exposed to H_2_O_2_ with/without GSH was assessed by qPCR (*n* = 3). (D) The migration of HUVECs with miR-139-5p mimics/inhibitors was evaluated using the scratch assay (scale bar: 200 μm) and (E) quantified by the ImageJ (*n* = 3). (F) The proliferation of HUVECs with miR-139-5p mimics/inhibitors was assessed by CCK8 assay (*n* = 3). (G and H) Apoptosis of HUVECs treated with miR-139-5p mimics or HUVECs treated with PA/PA + inhibitors were analyzed and quantified by flow cytometry (*n* = 3). (I) miR-139-5p expression in HUVECs after transfection was determined by qPCR (*n* = 3). *P < 0.05, **P < 0.01, ***P < 0.001, ****P < 0.0001.

Although some studies have shown that the upregulation of miR-139-5p is related to EC damage in diabetes (22), whether miR-139-5p mediates oxidative stress-induced damage to HUVECs is uncertain. Here, we observed a significant increase in miR-139-5p expression in HUVECs when exposed to H_2_O_2_, while pretreatment with the antioxidant GSH reversed this change (Fig. 2A, B, C), suggesting that oxidative stress induces the upregulation of miR-139-5p expression in ECs.

Next, we transfected HUVECs with miR-139-5p mimics to overexpress the miRNA- or PA-exposed HUVECs with miR-139-5p inhibitors to knock down its expression (Fig. 2I). The results showed that overexpression of miR-139-5p through mimics reduced the migration rate and proliferation viability of HUVECs, whereas the transfection of miR-139-5p inhibitors partially restored the migration and proliferation capacity that was diminished due to PA exposure (Fig. 2D, E, F). Moreover, overexpression of miR-139-5p in HUVECs increased the cells apoptosis rate, whereas downregulation of miR-139-5p significantly reduced PA-induced cells apoptosis (Fig. 2G and H). The above results indicated that miR-139-5p mediates oxidative stress-induced damage to HUVECs.

### miR-139-5p involved in HUVECs oxidative damage by targeting SOD1/GCLc

**Figure 3:**
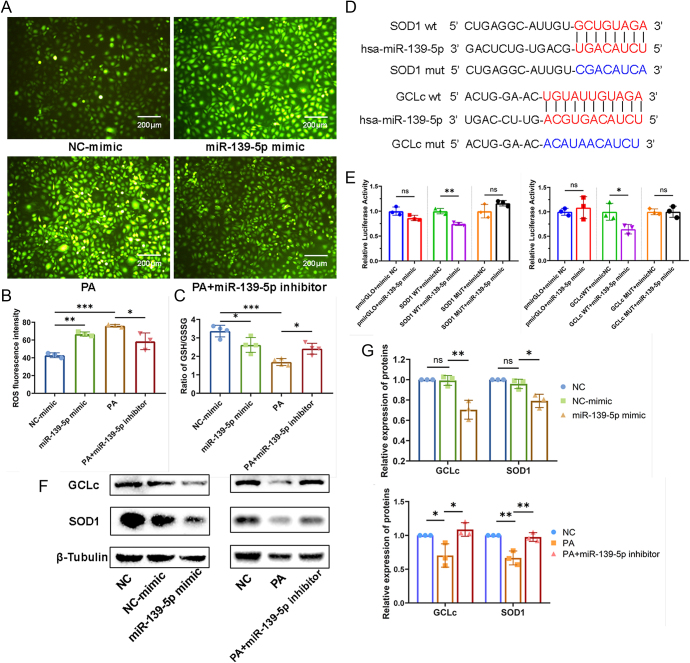
miR-139-5p regulates ROS production and SOD1 and GCLc in HUVECs. (A) ROS generation in HUVECs treated with miR-139-5p mimics or in HUVECs treated with PA or PA plus inhibitors was assessed by the DCFH-DA fluorescence (scale bar: 200 μm) and (B) quantified by the ImageJ (*n* = 3). (C) The GSH/GSSG ratio in HUVECs transfected with miR-139-5p mimic or miR-139-5p inhibitors in PA-medium (*n* = 4). (D and E) Dual-luciferase reporter assays were used to confirm that SOD1 and GCLc were targets of miR-139-5p (*n* = 3). (F) The expression of SOD1 and GCLc were detected by Western blot analysis and (G) quantified by the Image Lab (*n* = 3). *P < 0.05, **P < 0.01, ***P < 0.001, ns *P* > 0.05.

Furthermore, we observed that the introduction of miR-139-5p mimics significantly enhanced ROS production and decreased the GSH/GSSG ratio in HUVECs, while inhibition of miR-139-5p using inhibitors decreased ROS generation and partially restored the GSH/GSSG ratio in HUVECs that had been compromised by PA treatment in HUVECs (Fig. 3A, B, C). These findings suggest that miR-139-5p directly regulates the intracellular redox balance.

SOD1 and GCLc were identified as potential target genes of miR-139-5p through sequence-based prediction algorithms (Fig. 3D). The dual-luciferase reporter assay was performed and revealed that co-transfection of miR-139-5p mimic with the WT SOD1 (or WT GCLc) reporter plasmid significantly reduced relative luciferase activity compared to the NC mimic control. Whereas this suppressive effect was abolished when the predicted miR-139-5p binding site in the 3′UTR of target genes were mutated (Fig. 3E), indicating that miR-139-5p directly binds to the 3′UTR of SOD1 and GCLc. Then, we detected the expression of SOD1 and GCLc proteins by western blot. The results showed that the expression of SOD1 and GCLc in HUVECs treated with miR-139-5p mimics was significantly inhibited, while miR-139-5p inhibitors increased the PA-induced reduction of SOD1 and GCLc expression in HUVECs (Fig. 3F and G). These findings suggested that miR-139-5p directly target and inhibit SOD1/GCLc expression, which play crucial roles in EC oxidative stress.

### GLP-1 protects HUVECs from oxidative stress-induced damage via regulating miR-139-5p/SOD1/GCLc

**Figure 4:**
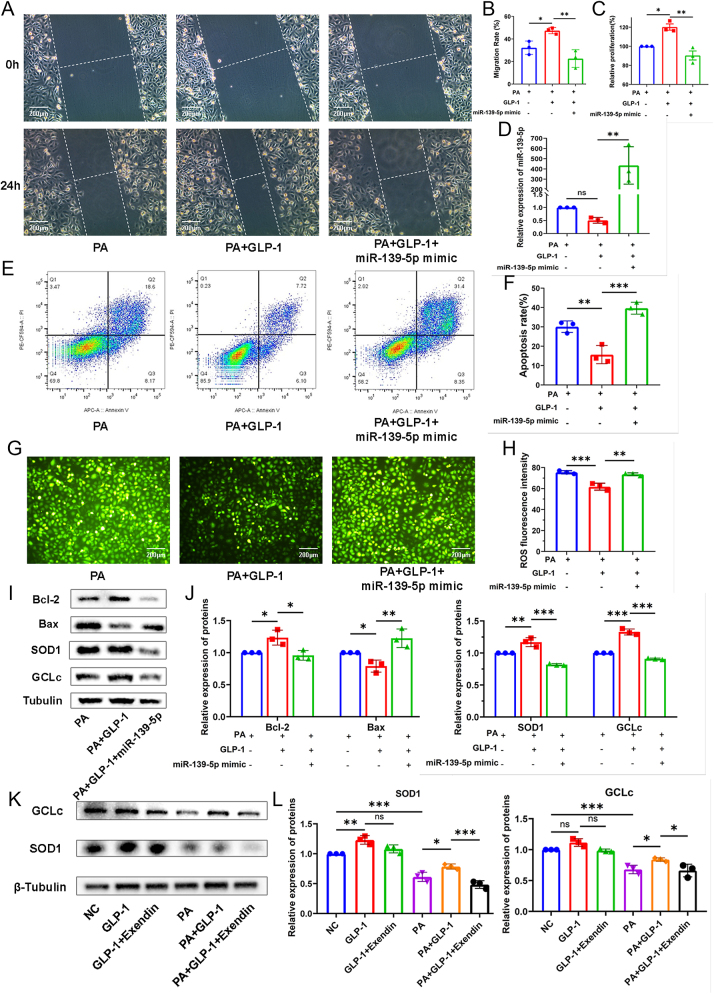
GLP-1 ameliorates oxidative stress damage induced by PA in HUVECs via miR-139-5p/SOD1 and GCLc. (A, B, C) Migration and proliferation of HUVECs treated with GLP-1 with/without miR-139-5p mimics were evaluated by scratch (*n* = 3) and CCK8 assay (*n* = 3) and quantified by the ImageJ. (D) The reversal of GLP-1-induced downregulation of miR-139-5p expression by miR-139-5p mimics was confirmed by qPCR (*n* = 3). (E and F) Representative images and apoptosis rate of HUVECs treated with GLP-1 in PA medium with/without miR-139-5p mimics were assessed by flow cytometry (*n* = 3). (G) ROS production was observed by DCFH-DA fluorescence (scale bar: 200 μm) and (H) quantified by the ImageJ (*n* = 3). (I and J) Western blot analysis showing changes in protein levels of Bcl-2, Bax, SOD1 and GCLc regulated by miR-139-5p mimics in response to GLP-1 treatment (*n* = 3). (K) Protein expression of SOD1 and GCLc measured by Western blot showing effects of exendin(9–39) against GLP-1 in normal or PA-treated HUVECs, (L) and quantified by the Image Lab (*n* = 3). ns *P* > 0.05, ***P < 0.001,**P < 0.01, *P < 0.05.

As observed above, miR-139-5p may be involved in mediating the protective effect of GLP-1 on ECs exposed to PA. To further clarify this, HUVECs were transfected with miR-139-5p mimics. The results showed that the miR-139-5p upregulation through mimics counteracted the GLP-1-induced improvement in migration and proliferation of HUVECs impaired by PA (Fig. 4A, B, C, D). Furthermore, the presence of miR-139-5p mimics abrogated the anti-apoptotic effect of GLP-1 on HUVECs exposed to PA (Fig. 4E and F). Similarly, miR-139-5p mimics transfection weakened GLP-1-induced Bcl-2 increase and Bax decrease in HUVECs exposed to PA (Fig. 4I and J). In addition, HUVECs transfected with miR-139-5p mimics showed an increase in ROS levels even with GLP-1 intervention (Fig. 4G and H). These results suggested that GLP-1 protects against PA-induced damage to HUVECs by regulating the expression of miR-139-5p.

Consistent with this, the expression of SOD1 and GCLc, target genes of miR-139-5p, significantly increased in HUVECs treated with GLP-1. However, upregulation of miR-139-5p by transfection markedly suppressed the increase in GCLc and SOD1 induced by GLP-1 treatment (Fig. 4I and J). Moreover, the upregulation of SOD1 and GCLc induced by GLP-1 were significantly attenuated upon addition of exendin(9–39) in PA-treated HUVECs (Fig. 4K and L), suggesting that GLP-1 receptor mediates the amelioration of GLP-1 on oxidative stress-induced HUVECs injury through miR-139-5p/SOD1/GCLc pathway.

## Discussion

GLP-1 receptor agonists (GLP-1RAs) and its analogs were shown to exert cardiovascular protective effects (24) and pro-angiogenic effects (25, 26) in diabetes, which cannot be solely attributed to the improved metabolic control of diabetes, but also recognized to be associated with a glucose-independent mechanism, which contributes to the amelioration of endothelial dysfunction (27). In the present study, we found that GLP-1(7–37) improved the migration, proliferation and angiogenic ability of HUVECs exposed to PA. In addition, it mitigated the excessive ROS generation, cell apoptosis and disrupted GSH/GSSG ratio induced by PA or HG. These findings align with previous studies showing that the upregulation of the GLP-1R enhanced the migration, adhesion, apoptosis and angiogenesis of late EPCs (28), and that GLP-1RAs reduce ROS in ECs by downregulating Nox4 and p47phox (29), upregulating uncoupling protein 2 (UCP2) (30) and increasing endoplasmic reticulum oxidoreductase (ERO1α) (31). Importantly, we observed a marked upregulation of miR-139-5p in HUVECs treated with HG or PA, which was significantly attenuated by GLP-1 pretreatment. Furthermore, we found that miR-139-5p upregulation increased intracellular ROS and decreased the GSH/GSSG ratio, and vice versa, suggesting that miR-139-5p is involved in the direct regulation of intracellular redox balance. The upregulation of miR-139-5p abrogated the protective effects of GLP-1 on HUVECs exposed to PA, and the GLP-1-induced downregulation of miR-139-5p was counteracted by the GLP-1 receptor antagonist exendin(9–39). These results indicate that the protective effect of GLP-1 on ECs exposed to PA/HG may be mediated through the regulation of miR-139-5p expression, primarily via the GLP-1 receptor. Collectively, these studies demonstrate that GLP-1/GLP-1RAs mitigate endothelial oxidative stress through diverse pathways. While other pathways focus on reducing ROS production or maintaining redox balance in specific cellular compartments, the miR-139-5p pathway enhances the overall antioxidant capacity of the cell.

Although miR-139-5p was initially extensively studied in tumor cells (32), its role in oxidative stress has attracted increasing attention. Recent researches suggested a potential pro-oxidant role of miR-139-5p. For example, miR-139-5p inhibition was shown to attenuate sodium butyrate-induced mitochondrial autophagy in bladder cancer cells by targeting Bmi-1 and reducing ROS production (33). Furthermore, Pajic *et al.* found that overexpression of miR-139-5p inhibits genes related to scavenging of ROS (20). Wang *et al.* (34) found that intrathecal injection of miR-139-5p angomir into spinal cord injured mice enhances mitochondrial ATP and ROS production. However, other scholars claimed that miR-139-5p might exert a protective role against oxidative damage in specific cells. Shao *et al.* (35) showed in retinal pigment epithelial cells that miR-139-5p reduces HG-induced oxidative stress and inflammation by regulating LIM-only factor 4. These contradictory findings may be due to the inconsistent oxidative stress pathways activated by miR-139-5p in different cell types and environment. Our research revealed that ROS induce the upregulation of miR-139-5p expression in ECs, which may contribute to a pro-oxidant effect in vascular ECs, mediating oxidative stress injury. This finding is consistent with other studies. For example, miR-139-5p has been shown to inhibit EC viability in acute myocardial infarction by targeting VEGFR-1, indirectly contributing to a pro-oxidant state (36). In addition, miR-139-5p can downregulate the expression of c-jun (22, 37), a component of the AP-1 transcription factor, which is involved in regulating the expression of antioxidant genes (38), and pro-angiogenic factors such as VEGF and PDGF. These findings highlight that the aberrant upregulation of miR-139-5p represents a pivotal target in the protective mechanism against oxidative damage in ECs.

Cu/ZnSOD (SOD1) and glutamate–cysteine ligase are key protective mechanisms against oxidative stress-induced endothelial disorders (39, 40). ROS scavenging via the upregulation of SOD1 can alleviate hyperglycemia-induced endothelial dysfunction (41) and improve endothelial-dependent relaxation (42). GCLc is involved in the synthesis of glutathione (GSH), a tripeptide that plays a crucial role in maintaining cellular redox balance. GCLc-deficient ECs exhibit elevated basal and stimulated ROS levels, leading to endothelial dysfunction (43). Our study is the first to demonstrate in HUVECs that miR-139-5p directly binds to and suppresses the expression of SOD1 and GCLc. Although previous studies have reported miR-139-5p′s targeting of SOD1 and GCLc in rat INS-1 cells on traditional Chinese medicine (44), our findings provide novel evidence for its regulatory role in human ECs and mediating oxidative stress-induced ECs injury. Abnormality of Keap1/Nrf2 is an important mechanism of impaired EC peroxidation in diabetes (45, 46). Although we did not directly detect Nrf2 in our study, previous research has shown that miR-139-5p can inhibit FoxO1, thereby regulating the Keap1/Nrf2 antioxidant pathway (47); it is plausible that miR-139-5p indirectly affects the Keap1/Nrf2 pathway. Moreover, our research revealed that the expression of miR-139-5p was significantly elevated in HUVECs exposed to H_2_O_2_, which was reversed by GSH, while GSH alone exerted no additional effect on basal miR-139-5p levels, these findings suggest a potential positive feedback loop between oxidative stress and miR-139-5p, amplifying EC damage. Therefore, miR-139-5p may involve a multitude of targets and pathways to mediate ECs damage in diabetes, especially its feedback enhancement of ROS production, may be an important mechanism of endothelial dysfunction in diabetes.

In addition, GLP-1R agonist exendin-4 has been shown to increase antioxidants (SOD-1 and glutathione peroxidase) to improve myocardial oxidative stress and ameliorates cardiac dysfunction in type 2 diabetes (48). GLP-1 has been also reported to restore GCLc expression in PC12 cells exposed to methylglyoxal through PI3K/Akt/mTOR signaling (49). Furthermore, it was found that the anti-apoptotic impact of liraglutide on the pancreas and INS-1 cells of diabetes rats was related to the inhibition of miR-139-5p/IRS1 expression (23). These studies suggest that the regulation of miR-139-5p could be a crucial mechanism through which GLP-1 exerts its antioxidant and anti-inflammatory effects. Our findings bridge this knowledge gap by demonstrating how GLP-1 ameliorates oxidative stress-induced ECs injury via miR-139-5p/SOD1/GCLc pathway. This miR-139-5p/SOD1/GCLc axis may serve as a crucial component of GLP-1-mediated antioxidant defense, appears to operate synergistically with the Nrf-2-dependent antioxidant pathway activation (46, 50), collectively constituting a multi-layered defense system against oxidative insult. These novel insight advances the understanding of how GLP-1 may protect endothelial dysfunction through miR-139-5p-mediated antioxidant reinforcement, potentially offering broader therapeutic implications for metabolic vascular disorders. Although we did not further validate the cardiovascular effects of GLP-1RAs and their association with miR-139-5p *in vivo*, it should be noted that clinically used GLP-1 receptor agonists (e.g., exenatide) and GLP-1 analogs (e.g., liraglutide and semaglutide) exhibit enhanced stability against serum degradation and prolonged half-life compared to native GLP-1(7–37). This pharmacokinetic advantage may translate to more potent antioxidant effects in conditions of endothelial oxidative stress. Supporting this notion, our experimental data demonstrate that exendin-4 (a representative GLP-1RA) achieved comparable or superior miR-139-5p suppression at a 20 nM concentration compared to 50 nM GLP-1(7–37), consistent with our hypothesis regarding the enhanced efficacy of clinically optimized analogs.

In conclusion, our study revealed that GLP-1 protects HUVECs from PA/HG-induced damage, at least in part, by regulating miR-139-5p/SOD1/GCLc in a GLP-1 receptor-dependent manner. This study provides further evidence for the vascular protective effects of GLP-1 intervention in diabetes by elucidating the molecular mechanisms mediated by the GLP-1 axis. Furthermore, our findings propose miR-139-5p as a potential therapeutic target for diseases driven by oxidative stress, including diabetes.

## Supplementary materials



## Declaration of interest

The authors declare that there is no conflict of interest that could be perceived as prejudicing the impartiality of the work reported.

## Funding

This work was supported by the National Natural Science Foundation of Chinahttps://doi.org/10.13039/501100001809 (grant number 81970724) and the Hainan Provincial Natural Science Foundation of Chinahttps://doi.org/10.13039/501100004761 (grant numbers 822MS201 and 822RC867).

## Author contribution statement

The present study was conceived, designed and supervised by Zhaohui Mo and Jing Xiong. Data acquisition was performed by Jiaqi Zhang, Jiake Mo and Ying Liu. Data analysis and interpretation was conducted by Jiaqi Zhang, Weian Tang, Xubiao Meng and Lanfang Fu. The manuscript was edited and reviewed by Jiaqi Zhang and Zhaohui Mo. All authors read and approved the final manuscript.

## Data availability

The datasets generated and analyzed during the current study are available from the corresponding author on reasonable request.
