# Supplementary figures and images for: Inhibition of microRNA-139-5p by glucagon-like peptide-1 ameliorates oxidative stress-induced vascular endothelial cell damage via targeting SOD1/GCLc
Source: Endocr Connect. 2025 Apr 29;14(5):e250022. doi: 10.1530/EC-25-0022 (PMC12060773; doi:10.1530/EC-25-0022)

## Supplementary Figure 1

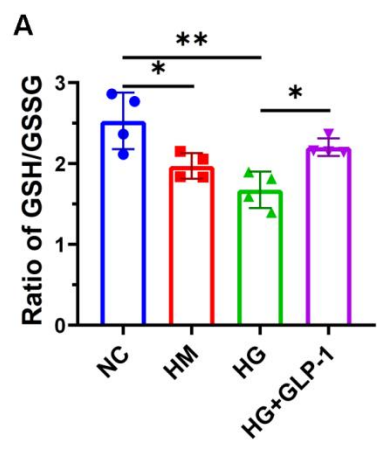

(a) Effects of GLP-1 on GSH/GSSG ratio in high glucose-induced HUVECs. \* $p < 0.05$ , \*\* $p < 0.01$  ( $n=4$ ).

Supplement: Supplementary file 1 [file supplementary_materials.pdf]
